# Synthesis and Antibacterial Evaluation of New *N*-acylhydrazone Derivatives from Dehydroabietic Acid

**DOI:** 10.3390/molecules17044634

**Published:** 2012-04-20

**Authors:** Wen Gu, Rongrong Wu, Shilong Qi, Chenhai Gu, Fanjunnan Si, Zhuhui Chen

**Affiliations:** College of Chemical Engineering, Nanjing Forestry University, Nanjing 210037, China

**Keywords:** *N*-acylhydrazone, dehydroabietic acid, synthesis, antibacterial activity

## Abstract

A series of new *N*-acylhydrazone derivatives were synthesized in good yields through the reactions of dehydroabietic acid hydrazide with a variety of substituted arylaldehydes. The structures of the synthesized compounds were confirmed by IR, ^1^H- and ^13^C-NMR, ESI-MS, elemental analysis and single crystal X-ray diffraction. From the crystal structure of compound **4l**, the C=N double bonds of these *N*-acylhydrazones showed (*E*)-configuration, while the NMR data of compounds **4a**–**q** indicated the existence of two rotamers for each compound in solution. The target compounds were evaluated for their antibacterial activities against four microbial strains. The result suggested that several compounds exhibited pronounced antibacterial activities. Particularly, compound **4p** exhibited good antibacterial activity against *Staphylococcus aureus* and *Bacillus subtilis* comparable to positive control. The possible antibacterial metabolism and the strategy for further optimization of this compound were also discussed.

## 1. Introduction

Microbial infections remain a pressing human health problem because of a combination of factors, including emerging infectious diseases and the rapid development of multi-drug resistance pathogens worldwide [1,2]. In spite of many antibiotics and chemotherapeutics available, there will still be a vital need to discover new and effective antimicrobial agents. In recent years, the *N*-acylhydrazone moiety has proved to be an important pharmacophore structure in pharmaceutical research [3]. These structures have received much attention due to their chemotherapeutic potential in the development of novel antimicrobial agents [4,5]. Some widely used antibacterial drugs such as furacilin, furazolidone and ftivazide are known to contain such kind of moieties [6]. In addition, many *N*-acylhydrazone derivatives have been reported to exhibit an array of biological activities such as antimalarial [7], antiviral [8], antitumor [9], anti-inflammatory [10], anticonvulsant [11], antidepressant [12] and vasodilative activities [13].

Naturally occurring diterpenoids with an abietane skeleton are often discovered and isolated from higher plants and their diverse bioactivities have been reported [14,15]. Among them, dehydroabietic acid (DHA, **1**) is one of the major tricyclic diterpenoid constituents of pine resin and can be readily obtained from commercial disproportionated rosin. DHA and its derivatives have exhibited a broad spectrum of biological activities such as antimicrobial, antitumor, antiviral, antioxidant, anti-inflammatory and gastroprotective activities [16,17,18,19,20,21], which indicate that the compound is a potentially useful starting material for the synthesis of industrially or pharmacologically important products. In view of these findings, it would be worthwhile to design, synthesize new derivatives of DHA bearing *N*-acylhydrazone moiety and evaluate their potential antimicrobial activities. In continuation of our previous study on new antimicrobial derivatives of resin acids [22], we report herein the synthesis and characterization of a series of new *N*-acylhydrazones starting from DHA. Their *in vitro* antibacterial activities against several test microbes are also presented.

## 2. Results and Discussion

### 2.1. Synthesis

**Scheme 1:**
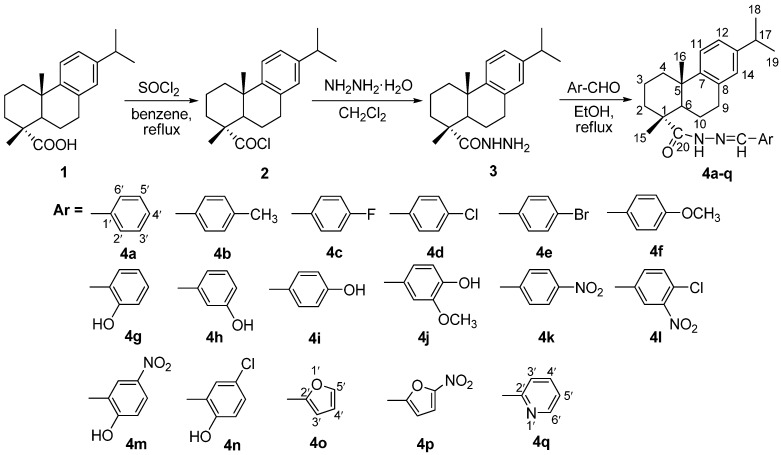
Synthetic route of hydrazone derivatives (**4a**–**q**) from dehydroabietic acid.

The synthetic procedures for the target compounds **4a**–**q** are outlined in Scheme 1. The key intermediate dehydroabietic acid hydrazide (**3**) was synthesized by hydrazination of dehydroabietate chloride (**2**), which was prepared by the reaction of dehydroabietic acid (**1**) with thionyl chloride [23]. In the former literature, the synthesis of hydrazides was usually carried out by hydrazination of methyl/ethyl esters of the corresponding carboxylic acids [24,25]. However, in this case, it was found that ethyl dehydroabietate could hardly be converted to the hydrazide **3**, possibly because of the low reactivity of the ester or the steric hindrance caused by the adjacent moieties of the molecule [26]. Alternatively the hydrazide could be synthesized in good yield from dehydroabietate chloride (**2**) with higher reactivity. Then the hydrazide **3** was condensed with different substituted aromatic aldehydes in refluxing ethanol to afford the corresponding *N*-acylhydrazones **4a**–**q** in good yield. 

### 2.2. Structural Analysis

The structures of the synthesized *N*-acylhydrazone derivatives were confirmed on the basis of IR, MS, NMR, elemental analysis and crystallographic methods. The IR spectra of **4a**–**q** exhibited in all cases N-H bands in the range 3,232–3,279 cm^−1^. The strong absorption bands at 1,646–1,658 cm^−1^ were due to the C=O stretch vibrations of the hydrazone moieties. The ESI-MS of compounds **4a**–**q** displayed, in all cases, quasimolecular ion peaks which confirmed their molecular weights. The ^1^H-NMR spectra of compounds **4a**–**q** were similar except for the aromatic protons. In a typical example, ^1^H-NMR spectra of **4a** showed a doublet at *δ* 1.22 (1.20) ppm corresponding to six methyl protons (H-18 and H-19) of the isopropyl group, together with a muliplet at *δ* 2.82 (2.78) ppm due to the vicinal CH proton (H-17). Two singlets at *δ *1.25 (1.24) and 1.40 (1.34) ppm could be assigned to methyl protons at C-16 and C-15, respectively. Resonance peaks attributing to eight aromatic protons in the two benzene rings appeared in the range *δ* 6.82–7.88 ppm. Among them, two doublets coupled with each other at *δ *7.00 (6.95) and 7.16 (7.08) ppm could be assigned to H-12 and H-11, respectively. The singlet at *δ *6.87 (6.82) ppm was due to the signal of H-14. Because of the symmetry of monosubstituted benzene ring, the signals of H-2′ and H-6′ appeared as a doublet at *δ *7.72 (7.88) ppm, while the signals at *δ *7.38–7.52 ppm could be assigned to H-3′, H-4′ and H-5′. The N=CH proton appeared as a singlet at *δ* 8.21 (8.03) ppm, and the amide NH proton resonated as a broad singlet at *δ* 8.83 (9.29) ppm. In the ^13^C-NMR spectra, the signal at *δ *23.9 (23.8) ppm could be assigned to C-18 and C-19. The carbon atoms of the two benzene rings resonated as ten pairs of absoption peaks because of the symmetry of substituent. In addition, the peaks at *δ *146.1 (145.8) and 175.2 (176.8) ppm could be attributed to the signals of C=N and C=O carbons, respectively. The ^1^H- and ^13^C-NMR signals of other *N*-acylhydrazones were assigned by comparing with those of **4a**.

**Figure 1:**
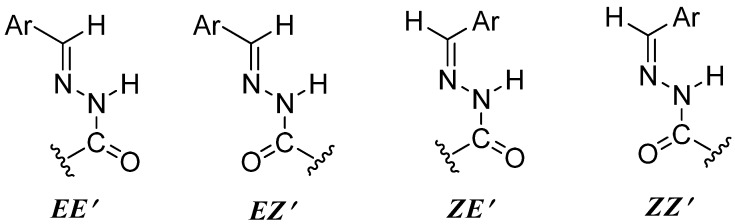
For possible forms of *N*-acylhydrazone derivatives.

Interestingly, in their ^1^H-NMR spectra, there were, if discriminable, two sets of signals observed for all groups, which resulted from the equilibrium and interconversion between rotamers (and/or configurational isomers) of each compound in solution [27,28]. It was known that *N*-acylhydrazones may exist in four possible forms in respect to (*E*/*Z*)-configurational isomers relative to the C=N double bonds and (*E*′/*Z*′)-rotamers caused by the inversion of amide bonds (Figure 1). However, the *E*/*Z* isomerization was not observed since these *N*-acylhydrazones exist primarily or completely in the (*E*)-configuration because of the steric hindrance relative to the moiety [29], which was supported by means of relative free energy calculations in a former report [30]. As for the characteristic peaks of *N*-acylhydrazones, the N=CH protons appeared as expected as two separate singlets in the region *δ* 7.71–8.58 ppm. The amide inversion also made the CONH proton present as two broad singlets in the range *δ* 8.71–10.18 ppm. 

**Figure 2:**
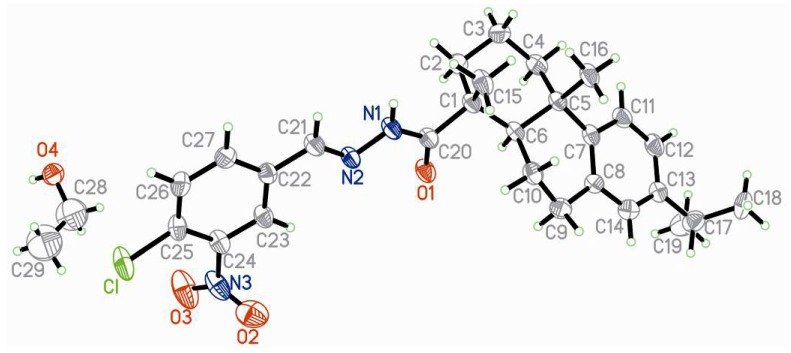
Crystal structure of compound **4l**.

The single crystal of the representative compound **4l** was recrystallized from ethanol for the X-ray diffraction to confirm the configuration. The crystal data of **4l** are presented in Table 1, and its ORTEP drawing with the atom numbering scheme is shown in Figure 2. In accordance with starting material dehydroabietic acid, the two cyclohexane ring A (C1~C6) and B (C5~C10) bearing chair and half-chair conformations, respectively, show a *trans* ring junction, with two methyl groups of C15 and C16 in the axial positions. The molecule displays an *E* configuration with respect to the N2=C21 double bond with a N1-N2-C21-C22 torsion angle of 179.5(3)°, and a *Z*′ conformation due to the amide bond with a N2-N1-C20-O1 torsion angle of 1.4(6)°.

**Table 1:** Crystal structure data for compound **4l**.

Empirical formula	C_29_H_38_ClN_3_O_4_	*F*(0 0 0)	1128
Formula weight	528.07	*θ* range for data collection (°)	1.93–25.38
Temperature (K)	293(2)	Max. and min. transmission	0.9674 and 0.9516
Crystal size (mm^3^)	0.30 × 0.20 × 0.20	Index ranges	0 ≤ *h* ≤ 15
Crystal system	Orthorhombic		0 ≤ *k* ≤ 15
Space group	*P*2_1_2_1_2_1_		−21 ≤ *l* ≤ 21
*a* (Å)	12.537(3)	Reflectons collected	5870
*b* (Å)	13.097(3)	Independent reflections	5374
*c* (Å)	17.855(4)	*R*_int_	0.0253
*α *(°)	90	Data/restraints/parameters	5374/3/328
*β *(°)	90	Goodness-of-fit on *F*^2^	1.001
*γ *(°)	90	*R*_1_, *wR*_2_ [*I* > 2*σ*(*I*)]	0.0592/0.1567
*V* (Å^3^)	2931.7(10)	*R*_1_, *wR*_2_ (all data)	0.0912/0.1783
*Z*	4	Flack parameter	−0.09(13)
*D_x _* (g/cm^−3^)	1.196	(Δρ)_max_ (eÅ^−3^)	0.520
*μ* (mm^−1^)	0.167	(Δρ)_min_ (eÅ^−3^)	−0.373

### 2.3. Antibacterial Activity

The synthesized compounds **3** and **4a**–**q** were evaluated for their antibacterial activities against four bacterial strains. The minimal inhibitory concentration (MIC) values of these compounds against the test microorganisms were listed in Table 2. Also included were the MICs of positive control amikacin. The result revealed that the MICs of the tested compounds differed greatly ranging from 1.9 to >100 μg/mL. It was found that compounds **4c**, **4d**, **4l**–**n** and **4p** exhibited significant activities (1.9–7.8 μg/mL) against Gram-positive bacteria (*S. aureus* and *B. subtilis*). Among them, compound **4p** showed the strongest activities against *S. aureus* and *B. subtilis *with MIC values (1.9 μg/mL) comparable to those of amikacin. Compounds **4l**, **4n** and **4p** also showed strong inhibitions (3.9–7.8 μg/mL) against the Gram-negative strains *Escherichia coli* and *Pseudomonas fluorescens*. In addition, compounds **3**, **4a**, **4e**, **4g**, **4h** and **4k** exhibited moderate activities (15.6–31.2 μg/mL) against at least one strain of the four bacteria, while **4b**, **4f**, **4i**, **4j**, **4o** and **4q** showed marginal or no inhibitions against the four strains. 

According to structure-activity relationships (SAR) studies, it could be concluded that different substitutions on aromatic rings affected drastically the antibacterial activities of the synthesized compounds. Compounds with electron-withdrawing substituents (halo and nitro) in the aromatic ring showed greater antibacterial activities than those with electron-donating groups (methyl and methoxy). The results of antibacterial assay indicated that the introduction of the halogen atom at 4′-position generally increased antibacterial activity, particularly F and Cl atoms, and compound **4n** with 3′-Cl substituent also showed marked antibacterial activity. Previous literatures revealed that the introduction of halogen atoms into the pharmacophore structure could, in many cases, be beneficial to antibacterial activity [31,32]. On the other hand, compounds **4l**, **4m** and **4p** with NO_2_ substituents exhibited good activities against the test bacteria, indicating that the introduction of NO_2_ group was essential for the antibacterial activity of these compounds. 

Some *N*-acylhydrazone derivatives with nitrophenyl and nitrofuranyl moieties have been reported to demonstrate pronounced antibacterial activities [33,34]. Among them, nifuroxazide, a notable example bearing nitrofuran moiety has been used as an intestinal antiseptic [34]. Previous investigations suggested that the possible antibacterial mechanism of these derivatives could be related to the reduction of the nitro group by microbial nitroreductase together with the formation of toxic intermediates such as nitro radical-anion, nitroso, hydroxylamine derivatives and/or reactive oxygen species [35,36]. However, enzymatic nitroreduction of nitrofurans has also been found in animal tissues, implying that toxic and mutagenic effects could also occur in mammalian cells [36]. A number of literatures demonstrated that some nitrofuran antibiotics such as nitrofurantoin and nitrofurazone showed mutagenic or carcinogenic effects on mammalian cells and tissues [36,37]. A prodrug strategy might give some clue to resolve this problem. The functional groups of a drug can be modified by chemical reactions such as esterification, amide formation, *etc.* to prepare a prodrug with better physico-chemical properties, which can be bioconverted to the parental drug *in vivo*. Some recent studies on the prodrugs of metronidazole and nitrofurazone suggested that the prodrug approach may increase the biological activity and decrease the genotoxicity of these nitro compounds by improving the physico-chemical properties [38]. In light of these findings, further derivatization of compound **4p** such as the introduction of various groups on the benzene ring, together with the in-depth SAR study and mutagenic assay are still required. 

**Table 2:** Antibacterial activities of compounds **4a**–**q**.

Test compounds	Minimum inhibitory concentration (μg/mL)			
	*S. aureus*	*B. subtilis*	*E. coli*	*P. fluorescens*
**3**	31.2	31.2	62.5	31.2
**4a**	31.2	62.5	>100	62.5
**4b**	>100	62.5	>100	>100
**4c**	7.8	3.9	31.2	>100
**4d**	7.8	15.6	31.2	62.5
**4e**	31.2	31.2	>100	>100
**4f**	>100	62.5	>100	>100
**4g**	62.5	15.6	31.2	62.5
**4h**	62.5	31.2	>100	>100
**4i**	>100	>100	>100	>100
**4j**	>100	62.5	>100	>100
**4k**	15.6	15.6	31.2	15.6
**4l**	3.9	3.9	15.6	7.8
**4m**	7.8	3.9	15.6	31.2
**4n**	3.9	7.8	15.6	7.8
**4o**	>100	62.5	>100	>100
**4p**	1.9	1.9	7.8	3.9
**4q**	62.5	>100	>100	>100
Amikacin	0.9	0.9	1.9	0.9

## 3. Experimental

### 3.1. General

Melting points were measured on an XT-4 apparatus (Taike Corp., Beijing, China) and were uncorrected. IR spectra were measured on a Nexus 870 FT-IR spectrometer. The ESI-MS spectra were recorded on a Mariner System 5304 mass spectrometer. ^1^H-NMR spectra were accomplished in CDCl_3_ on a Bruker AV-500 NMR spectrometer using TMS as internal standard. Elemental analyses were performed on a CHN-O-Rapid instrument and were within ±0.4% of the theoretical values. Reactions and the resulted products were monitored by TLC which was carried out on silica gel IB-F flexible sheets from Mallinckrodt Baker Inc., Germany and visualized in UV light (254 nm). Silica gel (300–400 mesh) for column chromatography was purchased from Qingdao Marine Chemical Factory, China. The reagents (chemicals), all being of A.R. grade, were purchased from Shanghai Chemical Reagent Company (Shanghai, China). The substituted aryl aldehydes were purchased from Alfa Aesar Co. (Tianjing, China). Disproportionated rosin was provided by Zhongbang Chemicals Co., Ltd. (Zhaoqing, China), from which dehydroabietic acid (**1**, 97%) was isolated according to the published method [39].

### 3.2. Crystallographic Studies

X-ray single-crystal diffraction data for compound **4l** was collected on an Enraf-Nonius CAD-4 diffractometer at 293(2) K using MoK_α_ radiation (*λ* = 0.71073 Å) by the *ω* scan mode. The structure was solved by direct methods using the SHELXS program of the SHELXTL package and refined by full-matrix least-squares methods with SHELXL [40]. All non-hydrogen atoms of compound **4l** were refined with anisotropic thermal parameters. All hydrogen atoms were generated theoretically onto the parent atoms and refined with riding model position parameters and fixed isotropic thermal factors. CCDC 848482 contains the supplementary crystallographic data for this paper. These data can be obtained free of charge via www.ccdc.cam.ac.uk/conts/retrieving.html (or from the CCDC, 12 Union Road, Cambridge CB2 1EZ, UK; Fax: +44-122-333-6033; E-Mail: deposit@ccdc.cam.ac.uk). 

### 3.3. Chemistry

#### 3.3.1. Dehydroabietic Acid Hydrazide (3)

SOCl_2_ (2.18 mL, 3.57 g, 0.03 mol) was added slowly to a solution of dehydroabietic acid (6 g, 0.02 mol) in benzene (20 mL). The mixture was then refluxed for 3 h. After cooling, the solvent and excess SOCl_2_ were removed *in vacuo* to yield dehydroabietate chloride (**2**) as a yellow oily product. The product was dissolved in CH_2_Cl_2_ (30 mL) and cooled to 0 °C, which was then added dropwise under stirring to a solution of hydrazine hydrate (12 mL, 80%) in CH_2_Cl_2_ (30 mL) at 0 °C. The mixture was stirred for 1–2 h, and the reaction progress was monitored by TLC. At the end of the reaction, the mixture was poured into cold water. The aqueous phase was extracted with CH_2_Cl_2_ (3 × 100 mL). The organic layer was combined, washed with water and brine, dried over anhydrous Na_2_SO_4_ and concentrated to give a crude product, which was subject to a silica gel column chromatography (petroleum ether-acetone 25:1, v/v) to give compound **3** as a whitish solid (4.02 g, 64%), m.p. 166–168 °C, IR (KBr, *υ*, cm^−1^): 3392, 3325, 1652, 1603, 1496, 1445, 1382, 1255, 832. ^1^H-NMR (CDCl_3_): 1.22 (d, *J* = 6.8 Hz, 6H, H-18 and H-19), 1.25 (s, 3H, CH_3_, H-16), 1.41 (s, 3H, CH_3_, H-15), 1.52–1.88 (m, 7H, H-2, H-3, H_a_-4 and H-10), 2.27 (d, *J* = 12.0 Hz, 1H, H-6), 2.36 (d, *J* = 12.9 Hz, 1H, H_e_-4), 2.82–2.90 (m, 3H, H-9 and H-17), 4.03 (brs, 2H, NH_2_), 6.89 (s, 1H, H-14), 7.01 (d, *J* = 8.1 Hz, 1H, H-12), 7.16 (d, *J* = 8.1 Hz, 1H, H-11), 8.39 (s, 1H, CONH). MS (ESI) *m*/*z* 315 ([M+H]^+^). Anal. Calcd for C_20_H_30_N_2_O: C, 76.39; H, 9.62, N, 8.91; found C, 76.51; H, 9.79; N, 8.78.

#### 3.3.2. Gerneral Procedure for the Syntheses of *N*-acylhydrazone Derivatives **4a**–**q**

To a solution of compound **3** (0.63 g, 2 mmol) in absolute EtOH (20 mL) was added 2 mmol of substituted aromatic aldehyde and 2 drops of glacial acetic acid. The mixture was refluxed for 2–3 h and the reaction was monitored by TLC. The mixture was then poured into cold water and left standing for another 4 h. The precipitate formed was filtered, washed with water and recrystalized from ethanol to afford compound **4**.

*Dehydroabietic acid 2-(phenylmethylene)hydrazide* (**4a**). White powder; yield 78%; m.p. 152–156 °C; IR (KBr, *υ*, cm^−1^): 3247, 3058, 2957, 2929, 2868, 1651, 1606, 1538, 1497, 1448, 1362, 1253, 821, 756. ^1^H-NMR (CDCl_3_, 500 MHz): 1.22 (1.20) (d, *J* = 7.0 Hz, 6H, H-18 and H-19), 1.25 (1.24) (s, 3H, CH_3_, H-16), 1.40 (1.34) (s, 3H, CH_3_, H-15), 1.50–1.95 (m, 7H, H-2, H-3, H_a_-4 and H-10), 2.27 (2.18) (d, *J* = 12.1 Hz, 1H, H-6), 2.34 (2.30) (d, *J* = 12.7 Hz, 1H, H_e_-4), 2.74–2.96 (m, 3H, H-9 and H-17), 6.87 (6.82) (s, 1H, H-14), 7.00 (6.95) (d, *J* = 8.1 Hz, 1H, H-12), 7.16 (7.08) (d, *J* = 8.3 Hz, 1H, H-11), 7.38–7.52 (m, 3H, H-3′, H-4′ and H-5′), 7.72 (7.88) (d, *J* = 8.2 Hz, 2H, H-2′ and H-6′), 8.21 (8.03) (s, 1H, N=CH), 8.83 (9.29) (s, 1H, CONH). ^13^C-NMR (CDCl_3_): 16.3 (15.8), 18.6 (18.4), 21.3 (21.2), 23.9 (23.8) (C-18 and C-19), 25.1 (25.0), 29.6 (29.8), 33.3 (33.2), 37.0 (36.9), 37.1 (37.2), 37.8 (37.7), 45.4 (45.2), 47.3 (47.1), 123.9 (123.8) (C-12), 124.1 (124.0) (C-11), 126.8 (126.7) (C-14), 127.8 (127.4) (C-3′ and C-5′), 129.2 (129.1) (C-2′ and C-6′), 130.6 (130.5) (C-4′), 134.5 (134.4) (C-8), 134.9 (134.8) (C-1′), 145.8 (145.7) (C-13), 146.1 (145.8) (C=N), 146.6 (146.5) (C-7), 175.2 (176.8) (C=O). MS (ESI) *m*/*z* 403 ([M+H]^+^). Anal. Calcd for C_27_H_34_N_2_O: C, 80.55; H, 8.51, N, 6.96; found C, 80.76; H, 8.39; N, 6.84. 

*Dehydroabietic acid 2-[(4-methylphenyl)methylene]hydrazide* (**4b**). White powder; yield 86%; m.p. 142–144 °C; IR (KBr, *υ*, cm^−1^): 3247, 2957, 2928, 2868, 1650, 1610, 1541, 1498, 1362, 1255, 819. ^1^H-NMR (CDCl_3_): 1.22 (1.21) (d, *J* = 7.0 Hz, 6H, H-18 and H-19), 1.25 (1.24) (s, 3H, CH_3_, H-16), 1.39 (1.34) (s, 3H, CH_3_, H-15), 1.50–1.95 (m, 7H, H-2, H-3, H_a_-4 and H-10), 2.25 (2.19) (d, *J* = 12.1 Hz, 1H, H-6), 2.33 (2.30) (d, *J* = 12.6 Hz, 1H, H_e_-4), 2.36 (2.44) (s, 3H, C_6_H_4_C*H*_3_), 2.78–2.95 (m, 3H, H-9 and H-17), 6.86 (6.80) (s, 1H, H-14), 7.00 (6.98) (d, *J* = 8.0 Hz, 1H, H-12), 7.14–7.19 (m, 3H, H-11, H-2′ and H-6′), 7.62 (7.77) (d, *J* = 7.0 Hz, 2H, H-3′ and H-5′), 8.15 (7.82) (s, 1H, N=CH), 8.76 (9.21) (s, 1H, CONH). ^13^C-NMR (CDCl_3_): 16.4 (15.8), 18.6 (18.4), 21.2 (21.1), 21.4 (21.5), 23.9 (23.8) (C-18 and C-19), 25.2 (25.0), 29.7 (29.8), 33.4 (33.2), 37.0 (36.9), 37.1 (37.2), 37.8 (37.7), 45.3 (45.2), 47.2 (46.8), 123.8 (123.7) (C-12), 124.0 (123.9) (C-11), 126.9 (126.8) (C-14), 127.6 (127.7) (C-2′ and C-6′), 129.3 (129.4) (C-3′ and C-5′), 131.0 (130.9) (C-1′), 134.6 (134.5) (C-8), 140.6 (140.5) (C-4′), 145.8 (145.7) (C-13), 146.8 (146.6) (C-7), 148.0 (148.4) (C=N), 174.4 (175.1) (C=O). MS (ESI) *m*/*z* 417 ([M+H]^+^). Anal. Calcd for C_28_H_36_N_2_O: C, 80.72; H, 8.71, N, 6.72; found C, 80.96; H, 8.62; N, 6.51.

*Dehydroabietic acid 2-[(4-fluorophenyl)methylene]hydrazide* (**4c**). White powder; yield 74%; m.p. 205–207 °C; IR (KBr, *υ*, cm^−1^): 3255, 2957, 2930, 2869, 1651, 1604, 1508, 1340, 1234, 835. ^1^H-NMR (CDCl_3_): 1.22 (1.21) (d, *J* = 7.0 Hz, 6H, H-18 and H-19), 1.25 (1.24) (s, 3H, CH_3_, H-16), 1.39 (1.34) (s, 3H, CH_3_, H-15), 1.51–1.90 (m, 7H, H-2, H-3, H_a_-4 and H-10), 2.26 (2.19) (d, *J* = 12.1 Hz, 1H, H-6), 2.34 (2.30) (d, *J* = 12.9 Hz, 1H, H_e_-4), 2.75–2.96 (m, 3H, H-9 and H-17), 6.87 (6.82) (s, 1H, H-14), 7.00 (6.95) (d, *J* = 8.0 Hz, 1H, H-12), 7.16 (7.08) (d, *J* = 8.0 Hz, 1H, H-11), 7.07 (7.21) (dd, *J* = 8.3, 8.1 Hz, 2H, H-3′ and H-5′), 7.71 (7.91) (dd, *J* = 8.3, 5.4 Hz, 2H, H-2′ and H-6′), 8.22 (8.05) (s, 1H, N=CH), 8.80 (9.28) (s, 1H, CONH). ^13^C-NMR (CDCl_3_): 16.4 (15.8), 18.6 (18.4), 21.3 (21.2), 23.9 (23.8) (C-18 and C-19), 25.2 (25.1), 29.7 (29.8), 33.4 (33.2), 37.0 (36.9), 37.1 (37.2), 37.8 (37.7), 45.3 (45.2), 47.3 (46.8), 115.6 (115.8) (^2^*J*_C-F_ = 21.2 Hz, C-3′ and C-5′), 123.8 (123.7) (C-12), 124.0 (123.9) (C-11), 126.8 (126.7) (C-14), 129.3 (129.4) (^3^*J*_C-F_ = 7.5 Hz, C-2′ and C-6′), 130.1 (130.0) (^4^*J*_C-F_ = 2.5 Hz, C-1′), 134.5 (134.4) (C-8), 145.8 (145.7) (C-13), 146.6 (146.5) (C-7), 146.9 (147.4) (C=N), 164.9 (162.9) (^1^*J*_C-F_ = 241.9 Hz, C-4′), 174.4 (174.8) (C=O). MS (ESI) *m*/*z* 421 ([M+H]^+^). Anal. Calcd for C_27_H_33_FN_2_O: C, 77.11; H, 7.91; N, 6.66; found C, 77.02; H, 8.06; N, 6.46. 

*Dehydroabietic acid 2-[(4-chlorophenyl)methylene]hydrazide *(**4d**). White powder; yield 70%; m.p. 137–139 °C; IR (KBr, *υ*, cm^−1^): 3243, 2957, 2929, 2869, 1651, 1597, 1538, 1490, 1361, 1254, 1089, 822. ^1^H-NMR (CDCl_3_): 1.22 (1.21) (d, *J* = 7.0 Hz, 6H, H-18 and H-19), 1.25 (1.24) (s, 3H, CH_3_, H-16), 1.39 (1.36) (s, 3H, CH_3_, H-15), 1.50–1.89 (m, 7H, H-2, H-3, H_a_-4 and H-10), 2.26 (2.10) (d, *J* = 12.1 Hz, 1H, H-6), 2.34 (2.26) (d, *J* = 12.9 Hz, 1H, H_e_-4), 2.79–2.92 (m, 3H, H-9 and H-17), 6.87 (6.81) (s, 1H, H-14), 7.00 (6.95) (d, *J* = 7.9 Hz, 1H, H-12), 7.16 (7.07) (d, *J* = 7.9 Hz, 1H, H-11), 7.35 (7.52) (d, *J* = 8.2 Hz, 2H, H-3′ and H-5′), 7.65 (7.82) (d, *J* = 7.8 Hz, 2H, H-2′ and H-6′), 8.21 (7.96) (s, 1H, N=CH), 8.84 (9.37) (s, 1H, CONH). ^13^C-NMR (CDCl_3_): 16.3 (15.8), 18.5 (18.4), 21.3 (21.2), 23.9 (23.8) (C-18 and C-19), 25.2 (25.1), 29.7 (29.8), 33.4 (33.2), 37.0 (36.9), 37.1 (37.2), 37.8 (37.7), 45.3 (45.2), 47.3 (47.0), 123.8 (123.7) (C-12), 124.0 (123.9) (C-11), 126.8 (126.7) (C-14), 128.6 (128.7) (C-3′ and C-5′), 128.8 (129.0) (C-2′ and C-6′), 132.3 (132.2) (C-1′), 134.5 (134.4) (C-8), 136.1 (136.0) (C-4′), 145.7 (145.6) (C-13), 146.6 (146.5) (C-7), 146.7 (146.9) (C=N), 175.0 (175.6) (C=O). MS (ESI) *m*/*z* 437 ([M+H]^+^). Anal. Calcd for C_27_H_33_ClN_2_O: C, 74.20; H, 7.61, N, 6.41; found C, 74.51; H, 7.36; N, 6.17. 

*Dehydroabietic acid 2-[(4-bromophenyl)methylene]hydrazide *(**4e**)*.* White powder; yield 65%; m.p. 115–117 °C; IR (KBr, *υ*, cm^−1^): 3270, 2958, 2929, 2869, 1651, 1606, 1538, 1486, 1382, 1253, 1069, 820. ^1^H-NMR (CDCl_3_): 1.22 (1.21) (d, *J* = 7.0 Hz, 6H, H-18 and H-19), 1.25 (1.23) (s, 3H, CH_3_, H-16), 1.39 (1.34) (s, 3H, CH_3_, H-15), 1.50–1.92 (m, 7H, H-2, H-3, H_a_-4 and H-10), 2.25 (2.19) (d, *J* = 12.1 Hz, 1H, H-6), 2.34 (2.31) (d, *J* = 13.0 Hz, 1H, H_e_-4), 2.73–2.95 (m, 3H, H-9 and H-17), 6.87 (6.82) (s, 1H, H-14), 6.99 (6.94) (d, *J* = 8.2 Hz, 1H, H-12), 7.16 (6.07) (d, *J* = 8.3 Hz, 1H, H-11), 7.51 (7.69) (d, *J* = 8.2 Hz, 2H, H-3′ and H-5′), 7.58 (7.75) (d, *J* = 8.0 Hz, 2H, H-2′ and H-6′), 8.21 (8.02) (s, 1H, N=CH), 8.83 (9.41) (s, 1H, CONH). ^13^C-NMR (CDCl_3_): 16.4 (15.8), 18.6 (18.4), 21.3 (21.2), 23.9 (23.8) (C-18 and C-19), 25.2 (25.1), 29.7 (29.8), 33.4 (33.2), 37.0 (36.9), 37.1 (37.2), 37.8 (37.3), 45.3 (45.2), 47.3 (46.9), 123.9 (123.8) (C-12), 124.1 (124.0) (C-11), 124.5 (124.2) (C-4′), 126.8 (126.7) (C-14), 128.8 (128.4) (C-2′ and C-6′), 131.8 (131.6) (C-3′ and C-5′), 132.8 (132.4) (C-1′), 134.5 (134.4) (C-8), 145.7 (145.6) (C-13), 146.6 (146.5) (C-7), 146.7 (147.1) (C=N), 174.5 (174.9) (C=O). MS (ESI) *m*/*z* 481, 483 ([M+H]^+^). Anal. Calcd for C_27_H_33_BrN_2_O: C, 67.35; H, 6.91, N, 5.82; found C, 67.12; H, 6.84; N, 5.96. 

*Dehydroabietic acid 2-[(4-methoxylphenyl)methylene]hydrazide *(**4f**). White powder; yield 89%; m.p. 130–132 °C; IR (KBr, *υ*, cm^−1^): 3242, 2957, 2930, 2868, 1646, 1606, 1510, 1463, 1381, 1252, 1169, 1033, 830. ^1^H-NMR (CDCl_3_): 1.22 (1.21) (d, *J* = 7.0 Hz, 6H, H-18 and H-19), 1.25 (1.24) (s, 3H, CH_3_, H-16), 1.39 (1.34) (s, 3H, CH_3_, H-15), 1.50–1.95 (m, 7H, H-2, H-3, H_a_-4 and H-10), 2.26 (2.18) (d, *J* = 11.5 Hz, 1H, H-6), 2.33 (2.29) (d, *J* = 12.8 Hz, 1H, H_e_-4), 2.78–2.96 (m, 3H, H-9 and H-17), 3.83 (3.89) (s, 3H, OCH_3_), 6.87 (6.88) (s, 1H, H-14), 6.90 (6.92) (d, *J* = 8.5 Hz, 2H, H-3′ and H-5′), 7.00 (7.05) (d, *J* = 7.0 Hz, 1H, H-12), 7.16 (7.14) (d, *J* = 7.0 Hz, 1H, H-11), 7.67 (7.79) (d, *J* = 7.8 Hz, 2H, H-2′ and H-6′), 8.12 (7.84) (s, 1H, N=CH), 8.83 (9.12) (s, 1H, CONH). ^13^C-NMR (CDCl_3_): 16.4 (15.8), 18.5 (18.4), 21.3 (21.2), 23.9 (23.8) (C-18 and C-19), 25.2 (25.1), 29.7 (29.8), 33.4 (33.2), 37.0 (36.9), 37.1 (37.2), 37.8 (37.7), 45.3 (45.2), 47.4 (47.1), 55.8 (55.6) (OCH_3_), 114.8 (114.7) (C-3′ and C-5′), 123.8 (123.7) (C-12), 124.0 (123.9) (C-11), 126.8 (126.7) (C-14), 128.0 (127.6) (C-1′), 131.0 (130.8) (C-2′ and C-6′), 134.6 (134.4) (C-8), 144.1 (144.5) (C=N), 145.8 (145.7) (C-13), 146.6 (146.5) (C-7), 161.2 (160.9) (C-4′), 175.7 (176.1) (C=O). MS (ESI) *m*/*z* 433 ([M+H]^+^). Anal. Calcd for C_28_H_36_N_2_O_2_: C, 77.74; H, 8.39, N, 6.48; found C, 77.42; H, 8.11; N, 6.68. 

*Dehydroabietic acid 2-[(2-hydroxyphenyl)methylene]hydrazide* (**4g**). White powder; yield 73%; m.p. 153–155 °C; IR (KBr, *υ*, cm^−1^): 3273, 2956, 2928, 2866, 1654, 1609, 1540, 1498, 1458, 1382, 1128, 820. ^1^H-NMR (CDCl_3_): 1.21 (1.20) (d, *J* = 7.0 Hz, 6H, H-18 and H-19), 1.26 (1.24) (s, 3H, CH_3_, H-16), 1.39 (1.36) (s, 3H, CH_3_, H-15), 1.50–1.88 (m, 7H, H-2, H-3, H_a_-4 and H-10), 2.25 (2.17) (d, *J* = 11.7 Hz, 1H, H-6), 2.34 (2.29) (d, *J* = 12.9 Hz, 1H, H_e_-4), 2.77–2.95 (m, 3H, H-9 and H-17), 6.85 (6.80) (s, 1H, H-14), 6.96 (7.04) (d, *J* = 8.2 Hz, 1H, H-3′), 7.00 (6.95) (d, *J* = 8.0 Hz, 1H, H-12), 7.08–7.19 (m, 2H, H-11 and H-5′), 7.52–7.67 (m, 2H, H-4′ and H-6′), 8.19 (7.83) (s, 1H, N=CH), 8.84 (9.36) (s, 1H, CONH), 9.91 (10.17) (s, 1H, 2′-OH). ^13^C-NMR (CDCl_3_): 16.3 (15.8), 18.5 (18.3), 21.4 (21.2), 23.8 (23.7) (C-18 and C-19), 25.1 (25.0), 29.7 (29.8), 33.3 (33.2), 37.0 (36.9), 37.1 (37.2), 37.8 (37.7), 45.4 (45.1), 47.3 (47.1), 116.9 (116.8) (C-3′), 119.8 (119.6) (C-5′), 120.1 (119.9) (C-1′), 123.9 (123.8) (C-12), 124.1 (124.0) (C-11), 126.8 (126.7) (C-14), 131.2 (130.9) (C-6′), 131.6 (131.3) (C-4′), 134.6 (134.5) (C-8), 145.8 (145.7) (C-13), 146.6 (146.5) (C-7), 146.8 (147.3) (C=N), 157.2 (156.9) (C-2′), 175.3 (175.8) (C=O). MS (ESI) *m*/*z* 419 ([M+H]^+^). Anal. Calcd for C_27_H_34_N_2_O_2_: C, 77.48; H, 8.19, N, 6.69; found C, 77.69; H, 8.37; N, 6.52. 

*Dehydroabietic acid 2-[(3-hydroxyphenyl)methylene]hydrazide* (**4h**). White powder; yield 86%; m.p. 135–137 °C; IR (KBr, *υ*, cm^−1^): 3276, 2957, 2930, 2869, 1652, 1602, 1539, 1455, 1382, 1362, 1230, 821. ^1^H-NMR (CDCl_3_): 1.21 (1.20) (d, *J* = 7.1 Hz, 6H, H-18 and H-19), 1.24 (1.23) (s, 3H, CH_3_, H-16), 1.37 (1.33) (s, 3H, CH_3_, H-15), 1.51–1.95 (m, 7H, H-2, H-3, H_a_-4 and H-10), 2.26 (2.18) (d, *J* = 11.5 Hz, 1H, H-6), 2.32 (2.21) (d, *J* = 12.3 Hz, 1H, H_e_-4), 2.78–2.92 (m, 3H, H-9 and H-17), 6.84 (6.80) (s, 1H, H-14), 6.88 (6.90) (d, *J* = 7.9 Hz, 1H, H-4′), 6.91–7.04 (m, 2H, H-12 and H-5′), 7.13-7.18 (m, 2H, H-11and H-6′), 7.29 (7.39) (s, 1H, H-2′), 8.01 (7.73) (s, 1H, N=CH), 8.71 (9.09) (s, 1H, CONH), 9.37 (9.72) (s, 1H, 3′-OH). ^13^C-NMR (CDCl_3_): 16.3 (15.8), 18.5 (18.4), 21.3 (21.2), 23.9 (23.8) (C-18 and C-19), 25.2 (25.1), 29.7 (29.8), 33.4 (33.2), 37.0 (36.9), 37.1 (37.2), 37.8 (37.7), 45.4 (45.2), 47.3 (47.1), 114.5 (114.2) (C-2′), 118.2 (118.1) (C-4′), 121.8 (121.6) (C-6′), 123.8 (123.7) (C-12), 124.0 (123.9) (C-11), 126.8 (126.7) (C-14), 130.6 (130.5) (C-5′), 134.5 (134.3) (C-8), 138.7 (138.5) (C-1′), 145.7 (145.6) (C-13), 146.6 (146.5) (C-7), 146.9 (147.2) (C=N), 158.6 (158.4) (C-3′), 175.5 (176.0) (C=O). MS (ESI) *m*/*z* 419 ([M+H]^+^). Anal. Calcd for C_27_H_34_N_2_O_2_: C, 77.48; H, 8.19, N, 6.69; found C, 77.70; H, 8.01; N, 6.45. 

*Dehydroabietic acid 2-[(4-hydroxyphenyl)methylene]hydrazide *(**4i**). White powder; yield 79%; m.p. 297–298 °C; IR (KBr, *υ*, cm^−1^): 3279, 2958, 2932, 2866, 1653, 1612, 1536, 1452, 1380, 1367, 1227, 830. ^1^H-NMR (CDCl_3_): 1.22 (1.21) (d, *J* = 7.1 Hz, 6H, H-18 and H-19), 1.25 (1.24) (s, 3H, CH_3_, H-16), 1.38 (1.34) (s, 3H, CH_3_, H-15), 1.52–1.89 (m, 7H, H-2, H-3, H_a_-4 and H-10), 2.26 (2.19) (d, *J* = 11.2 Hz, 1H, H-6), 2.36 (2.32) (d, *J* = 12.8 Hz, 1H, H_e_-4), 2.80–2.98 (m, 3H, H-9 and H-17), 6.87 (6.81) (s, 1H, H-14), 6.95 (7.05) (d, *J* = 8.1 Hz, 2H, H-3′ and H-5′), 6.99 (6.94) (d, *J* = 8.2 Hz, 1H, H-12), 7.16 (7.08) (d, *J* = 8.0 Hz, 1H, H-11), 7.63 (7.79) (d, *J* = 8.3 Hz, 2H, H-2′ and H-6′), 8.12 (7.88) (s, 1H, N=CH), 8.79 (9.12) (s, 1H, CONH), 9.87 (9.58) (s, 1H, 4′-OH). ^13^C-NMR (CDCl_3_): 16.3 (15.8), 18.5 (18.4), 21.3 (21.2), 23.9 (23.8) (C-18 and C-19), 25.2 (25.1), 29.7 (29.8), 33.3 (33.2), 37.0 (36.9), 37.1 (37.2), 37.8 (37.7), 45.3 (45.2), 47.3 (46.9), 116.2 (116.0) (C-3′ and C-5′), 123.8 (123.7) (C-12), 124.0 (123.9) (C-11), 126.1 (126.0) (C-1′), 126.8 (126.7) (C-14), 130.6 (130.4) (C-2′ and C-6′), 134.5 (134.3) (C-8), 144.8 (145.2) (C=N), 145.7 (145.6) (C-13), 146.7 (146.5) (C-7), 160.8 (160.5) (C-4′), 175.1 (175.6) (C=O). MS (ESI) *m*/*z* 419 ([M+H]^+^). Anal. Calcd for C_27_H_34_N_2_O_2_: C, 77.48; H, 8.19, N, 6.69; found C, 77.31; H, 8.36; N, 6.82. 

*Dehydroabietic acid 2-[(4-hydroxy-3-methoxylphenyl)methylene]*hydrazide (**4j**). White powder; yield 92%; m.p. 138–140 °C; IR (KBr, *υ*, cm^−1^): 3252, 2958, 2930, 2875, 1651, 1600, 1538, 1455, 1384, 1270, 1052, 820, 742. ^1^H-NMR (CDCl_3_): 1.22 (1.21) (d, *J* = 6.8 Hz, 6H, H-18 and H-19), 1.25 (1.24) (s, 3H, CH_3_, H-16), 1.39 (1.34) (s, 3H, CH_3_, H-15), 1.53–1.95 (m, 7H, H-2, H-3, H_a_-4 and H-10), 2.27 (2.18) (d, *J* = 10.4 Hz, 1H, H-6), 2.34 (2.30) (d, *J* = 12.8 Hz, 1H, H_e_-4), 2.80–2.92 (m, 3H, H-9 and H-17), 3.93 (3.99) (s, 3H, OCH_3_), 5.89 (brs, 1H, OH), 6.86 (6.80) (s, 1H, H-14), 6.89 (7.04) (d, *J* = 8.1 Hz, 1H, H-5′), 6.98 (7.12) (d, *J* = 8.4 Hz, 1H, H-6′), 7.00 (6.95) (d, *J* = 8.2 Hz, 1H, H-12), 7.17 (7.06) (d, *J* = 8.2 Hz, 1H, H-11), 7.50 (7.42) (s, 1H, H-2′), 8.05 (7.71) (s, 1H, N=CH), 8.76 (9.08) (s, 1H, CONH). ^13^C-NMR (CDCl_3_): 16.3 (15.9), 18.6 (18.5), 21.2(21.1), 23.9 (23.8) (C-18 and C-19), 25.1 (25.0), 29.7 (29.8), 33.4 (33.2), 37.0 (36.9), 37.1 (37.2), 37.8 (37.4), 45.3 (45.2), 47.1 (46.7), 56.1 (56.0) (OCH_3_), 107.7 (107.6) (C-2′), 114.0 (114.2) (C-5′), 123.5 (123.4) (C-6′), 123.8 (123.7) (C-12), 124.0 (123.9) (C-11), 126.1 (126.2) (C-1′), 126.8 (126.7) (C-14), 134.6 (134.4) (C-8), 145.7 (145.6) (C-13), 146.8 (146.7) (C-7), 147.2 (147.5) (C=N), 148.2 (148.0) (C-3′), 148.3 (148.4) (C-4′), 174.7 (175.2) (C=O). MS (ESI) *m*/*z* 449 ([M+H]^+^). Anal. Calcd for C_28_H_36_N_2_O_3_: C, 74.97; H, 8.09, N, 6.24; found C, 74.73; H, 7.96; N, 6.45. 

*Dehydroabietic acid 2-[(4-nitrophenyl)methylene]hydrazide *(**4k**). Yellow powder; yield 71%; m.p. 148–150 °C; IR (KBr, *υ*, cm^−1^): 3255, 2956, 2927, 2869, 1657, 1602, 1523, 1473, 1382, 1249, 1172, 830. ^1^H-NMR (CDCl_3_): 1.22 (1.21) (d, *J* = 6.8 Hz, 6H, H-18 and H-19), 1.26 (1.24) (s, 3H, CH_3_, H-16), 1.41 (1.38) (s, 3H, CH_3_, H-15), 1.50–1.91 (m, 7H, H-2, H-3, H_a_-4 and H-10), 2.24 (2.17) (d, *J* = 11.6 Hz, 1H, H-6), 2.35 (2.30) (d, *J* = 13.2 Hz, 1H, H_e_-4), 2.75–2.96 (m, 3H, H-9 and H-17), 6.88 (6.82) (s, 1H, H-14), 7.01 (6.95) (d, *J* = 8.1 Hz, 1H, H-12), 7.17 (7.07) (d, *J* = 8.1 Hz, 1H, H-11), 7.87 (8.07) (d, *J* = 8.5 Hz, 2H, H-3′ and H-5′), 8.23 (8.39) (d, *J* = 8.5, 2H, H-2′ and H-6′), 8.44 (8.32) (s, 1H, N=CH), 9.31 (9.87) (s, 1H, CONH). ^13^C-NMR (CDCl_3_): 16.3 (15.8), 18.5 (18.4), 21.3 (21.2), 23.9 (23.8) (C-18 and C-19), 25.1 (25.0), 29.7 (29.8), 33.4 (33.2), 37.0 (36.8), 37.1 (37.2), 37.8 (37.7), 45.4 (45.3), 47.5 (46.9), 123.8 (123.6) (C-2′ and C-6′), 123.9 (123.7) (C-12), 124.0 (124.1) (C-11), 126.8 (126.7) (C-14), 127.9 (127.8) (C-3′ and C-5′), 134.3 (134.4) (C-8), 140.0 (140.1) (C-1′), 145.0 (145.5) (C=N), 145.9 (145.8) (C-13), 146.6 (146.5) (C-7), 148.5 (148.6) (C-4′), 174.6 (175.4) (C=O). MS (ESI) *m*/*z* 448 ([M+H]^+^). Anal. Calcd for C_27_H_33_N_3_O_3_: C, 72.46; H, 7.43, N, 9.39; found C, 72.79; H, 7.18; N, 9.12. 

*Dehydroabietic acid 2-[(4-chloro-3-nitrophenyl)methylene]hydrazide* (**4l**). Yellow crystals; yield 73%; m.p. 248–250 °C; IR (KBr, *υ*, cm^−1^): 3232, 2957, 2923, 2869, 1658, 1605, 1537, 1473, 1383, 1350, 1246, 1128, 822. ^1^H-NMR (CDCl_3_): 1.22 (1.21) (d, *J* = 7.1 Hz, 6H, H-18 and H-19), 1.25 (1.24) (s, 3H, CH_33_, H-16), 1.40 (1.36) (s, 3H, CH_3_, H-15), 1.52–1.90 (m, 7H, H-2, H-3, H_a_-4 and H-10), 2.24 (2.10) (d, *J* = 12.0 Hz, 1H, H-6), 2.35 (2.28) (d, *J* = 13.0 Hz, 1H, H_e_-4), 2.72–2.95 (m, 3H, H-9 and H-17), 6.87 (6.81) (s, 1H, H-14), 7.00 (6.94) (d, *J* = 7.9 Hz, 1H, H-12), 7.17 (7.07) (d, *J* = 8.2 Hz, 1H, H-11), 7.55 (7.76) (d, *J* = 8.4 Hz, 1H, H-5′), 7.89 (8.03) (d, *J* = 8.1 Hz, 1H, H-6′), 8.13 (8.36) (s, 1H, H-2′), 8.44 (8.27) (s, 1H, N=CH), 9.82 (10.18) (s, 1H, CONH). ^13^C-NMR (CDCl_3_): 16.4 (15.8), 18.6 (18.4), 21.2 (21.1), 23.9 (23.8) (C-18 and C-19), 25.2 (25.1), 29.7 (29.8), 33.4 (33.2), 37.0 (36.9), 37.1 (37.2), 37.8 (37.4), 45.3 (45.2), 47.2 (46.8), 123.5 (123.2) (C-2′), 123.8 (123.7) (C-12), 124.0 (123.9) (C-11), 126.3 (125.9) (C-1′), 126.8 (126.7) (C-14), 129.2 (129.0) (C-5′), 131.9 (131.5) (C-6′), 134.4 (134.2) (C-4′), 134.6 (134.5) (C-8), 145.7 (145.6) (C-13), 146.6 (146.5) (C-7), 146.8 (147.2) (C=N), 147.9 (147.7) (C-3′), 174.3 (174.7) (C=O). MS (ESI) *m*/*z* 482 ([M+H]^+^). Anal. Calcd for C_27_H_32_ClN_3_O_3_: C, 67.28; H, 6.69, N, 8.72; found C, 67.43; H, 6.91; N, 8.51. 

*Dehydroabietic acid 2-[(2-hydroxy-5-nitrophenyl)methylene]hydrazide* (**4m**). Yellow powder; yield 68%; m.p. 158–160 °C; IR (KBr, *υ*, cm^−1^): 3269, 2959, 2928, 2869, 1655, 1610, 1513, 1478, 1383, 1340, 1247, 1174, 822. ^1^H-NMR (CDCl_3_): 1.22 (1.21) (d, *J* = 6.8 Hz, 6H, H-18 and H-19), 1.25 (1.24) (s, 3H, CH_3_, H-16), 1.41 (1.35) (s, 3H, CH_3_, H-15), 1.50–1.92 (m, 7H, H-2, H-3, H_a_-4 and H-10), 2.18 (2.10) (d, *J* = 11.8 Hz, 1H, H-6), 2.36 (2.24) (d, *J* = 13.0 Hz, 1H, H_e_-4), 2.80–2.92 (m, 3H, H-9 and H-17), 6.87 (6.82) (s, 1H, H-14), 7.01 (6.95) (d, *J* = 7.8 Hz, 1H, H-12), 7.06 (7.10) (d, *J* = 8.9 Hz, 1H, H-3′), 7.16 (7.08) (d, *J* = 8.2 Hz, 1H, H-11), 8.15 (8.04) (s, 1H, H-6′), 8.17 (8.09) (d, *J* = 8.0 Hz, 1H, H-4′), 8.51 (8.48) (s, 1H, N=CH), 9.03 (9.76) (s, 1H, CONH), 10.02 (s, 1H, 2′-OH). ^13^C-NMR (CDCl_3_): 16.2 (15.8), 18.4 (18.3), 21.3 (21.2), 23.9 (23.8) (C-18 and C-19), 25.1 (25.0), 29.6 (29.7), 33.3 (33.2), 37.0 (36.9), 37.1 (37.2), 37.7 (37.6), 45.4 (45.2), 47.3 (47.1), 117.5 (117.4) (C-3′), 117.8 (117.9) (C-1′), 123.7 (123.6) (C-12), 123.9 (123.8) (C-11), 126.4 (126.3) (C-6′), 126.7 (126.6) (C-14), 126.8 (127.0) (C-4′), 134.2 (134.1) (C-8), 140.0 (139.9) (C-5′), 145.8 (145.7) (C-13), 146.5 (146.3) (C-7), 147.7 (148.1) (C=N), 163.7 (163.8) (C-2′), 175.3 (175.8) (C=O). MS (ESI) *m*/*z* 464 ([M+H]^+^). Anal. Calcd for C_27_H_33_N_3_O_4_: C, 69.95; H, 7.18, N, 9.06; found C, 69.78; H, 7.26; N, 8.92. 

*Dehydroabietic acid 2-[(5-chloro-2-hydroxyphenyl)methylene]hydrazide *(**4n**). White powder; yield 73%; m.p. 133–134 °C; IR (KBr, *υ*, cm^−1^): 3269, 2957, 2930, 2869, 1650, 1611, 1530, 1478, 1383, 1345, 1272, 952, 821. ^1^H-NMR (CDCl_3_): 1.22 (1.21) (d, *J* = 6.8 Hz, 6H, H-18 and H-19), 1.25 (1.24) (s, 3H, CH_3_, H-16), 1.40 (1.36) (s, 3H, CH_3_, H-15), 1.48–1.90 (m, 7H, H-2, H-3, H_a_-4 and H-10), 2.17 (2.08) (d, *J* = 11.8 Hz, 1H, H-6), 2.35 (2.30) (d, *J* = 12.9 Hz, 1H, H_e_-4), 2.72–2.95 (m, 3H, H-9 and H-17), 6.88 (6.81) (s, 1H, H-14), 6.93 (6.96) (d, *J* = 8.8 Hz, 1H, H-3′), 7.01 (7.07) (d, *J* = 8.0 Hz, 1H, H-12), 7.14–7.25 (m, 3H, H-11, H-4′ and H-6′), 8.37 (8.18) (s, 1H, N=CH), 8.83 (8.68) (s, 1H, 2′-OH), 9.84 (10.13) (s, 1H, CONH). ^13^C-NMR (CDCl_3_): 16.3 (15.8), 18.5 (18.4), 21.3 (21.2), 23.9 (23.8) (C-18 and C-19), 25.1 (25.0), 29.6 (29.8), 33.4 (33.2), 37.0 (36.9), 37.1 (37.2), 37.8 (37.7), 45.5 (45.3), 47.3 (46.9), 118.6 (118.5) (C-3′), 118.7 (118.8) (C-1′), 123.7 (123.6) (C-5′), 123.9 (123.8) (C-12), 124.0 (124.1) (C-11), 126.9 (126.8) (C-14), 129.7 (129.6) (C-6′), 131.3 (131.1) (C-4′), 134.4 (134.5) (C-8), 145.9 (145.8) (C-13), 146.6 (146.5) (C-7), 148.9 (149.2) (C=N), 157.0 (157.1) (C-2′), 174.7 (175.2) (C=O). MS (ESI) *m*/*z* 453 ([M+H]^+^). Anal. Calcd for C_27_H_33_ClN_2_O_2_: C, 71.58; H, 7.34, N, 6.18; found C, 71.79; H, 7.51; N, 6.02. 

*Dehydroabietic acid 2-[(furan-2-yl)methylene]hydrazide *(**4o**). White powder; yield 77%; m.p. 127–129 °C; IR (KBr, *υ*, cm^−1^): 3247, 2957, 2929, 2868, 1652, 1610, 1532, 1474, 1387, 1248, 1134, 952, 820, 742. ^1^H-NMR (CDCl_3_): 1.21 (1.20) (d, *J* = 7.0 Hz, 6H, H-18 and H-19), 1.26 (1.24) (s, 3H, CH_3_, H-16), 1.45 (1.38) (s, 3H, CH_3_, H-15), 1.48–1.90 (m, 7H, H-2, H-3, H_a_-4 and H-10), 2.23 (2.16) (d, *J* = 11.9 Hz, 1H, H-6), 2.33 (2.29) (d, *J* = 12.0 Hz, 1H, H_e_-4), 2.75–2.95 (m, 3H, H-9 and H-17), 6.60 (6.48) (dd, *J* = 3.6, 1.8 Hz, 1H, H-4′), 6.86 (6.81) (s, 1H, H-14), 6.73 (6.58) (dd, *J* = 3.6, 0.8 Hz, 1H, H-3′), 6.99 (6.94) (d, *J* = 8.0 Hz, 1H, H-12), 7.16 (7.06) (d, *J* = 8.1 Hz, 1H, H-11), 7.58 (7.52) (dd, *J* = 1.8, 0.8 Hz, 1H, H-5′), 8.45 (7.89) (s, 1H, N=CH), 9.67 (10.11) (s, 1H, CONH). ^13^C-NMR (CDCl_3_): 16.4 (15.8), 18.5 (18.4), 21.4 (21.2), 23.9 (23.8) (C-18 and C-19), 25.2 (25.1), 29.7 (29.8), 33.4 (33.2), 37.0 (36.9), 37.1 (37.2), 37.8 (37.7), 45.5 (45.3), 47.3 (46.8), 112.7 (112.5) (C-3′), 113.4 (113.2) (C-4′), 123.7 (123.6) (C-12), 123.9 (123.8) (C-11), 126.8 (126.7) (C-14), 134.2 (134.1) (C-8), 137.2 (137.6) (C=N), 145.4 (145.2) (C-5′), 145.8 (145.7) (C-13), 146.6 (146.5) (C-7), 149.0 (149.6) (C-2′), 175.3 (175.9) (C=O). MS (ESI) *m*/*z* 393 ([M+H]^+^). Anal. Calcd for C_25_H_32_N_2_O_2_: C, 76.49; H, 8.22, N, 7.14; found C, 76.33; H, 8.05; N, 7.36. 

*Dehydroabietic acid 2-[(5-nitrofuran-2-yl)methylene]hydrazide *(**4p**). White powder; yield 77%; m.p. 127–129 °C; IR (KBr, *υ*, cm^−1^): 3256, 2959, 2932, 2866, 1651, 1607, 1522, 1465, 1382, 1241, 1136, 823, 755; ^1^H-NMR (CDCl_3_): 1.21 (1.20) (d, *J* = 6.8 Hz, 6H, H-18 and H-19), 1.25 (1.24) (s, 3H, CH_3_, H-16), 1.42 (1.36) (s, 3H, CH_3_, H-15), 1.46–1.92 (m, 7H, H-2, H-3, H_a_-4 and H-10), 2.21 (2.15) (d, *J* = 11.7 Hz, 1H, H-6), 2.32 (2.28) (d, *J* = 12.6 Hz, 1H, H_e_-4), 2.78–2.90 (m, 3H, H-9 and H-17), 6.86 (6.80) (s, 1H, H-14), 7.00 (6.94) (d, *J* = 8.1 Hz, 1H, H-12), 7.28 (7.42) (d, *J* = 3.8 Hz, 1H, H-3′), 7.17 (7.05) (d, *J* = 8.1 Hz, 1H, H-11), 7.72 (7.85) (d, *J* = 3.8 Hz, 1H, H-4′), 8.39 (8.11) (s, 1H, N=CH), 9.51 (9.85) (s, 1H, CONH). ^13^C-NMR (CDCl_3_): 16.3 (15.8), 18.5 (18.4), 21.3 (21.2), 23.9 (23.8) (C-18 and C-19), 25.2 (25.1), 29.7 (29.8), 33.4 (33.2), 37.0 (36.9), 37.1 (37.2), 37.7 (37.6), 45.4 (45.3), 47.3 (47.0), 114.6 (114.5) (C-3′), 115.1 (115.0) (C-4′), 123.8 (123.7) (C-12), 124.0 (123.8) (C-11), 126.8 (126.7) (C-14), 134.3 (134.2) (C-8), 137.8 (138.2) (C=N), 145.8 (145.7) (C-13), 146.6 (146.5) (C-7), 151.5 (151.2) (C-5′), 152.0 (151.8) (C-2′), 175.1 (175.6) (C=O). MS (ESI) *m*/*z* 438 ([M+H]^+^). Anal. Calcd for C_25_H_31_N_3_O_4_: C, 68.63; H, 7.14, N, 9.60; found C, 68.48; H, 7.21; N, 9.45. 

*Dehydroabietic acid 2-[(pyridin-2-yl)methylene]hydrazide *(**4q**). White powder; yield 74%; m.p. 238–240 °C; IR (KBr, *υ*, cm^−1^): 3260, 2957, 2930, 2869, 1652, 1611, 1528, 1470, 1382, 1271, 1132, 820, 776; ^1^H-NMR (CDCl_3_): 1.22 (1.21) (d, *J* = 7.0 Hz, 6H, H-18 and H-19), 1.25 (1.24) (s, 3H, CH_3_, H-16), 1.41 (1.36) (s, 3H, CH_3_, H-15), 1.48–1.95 (m, 7H, H-2, H-3, H_a_-4 and H-10), 2.22 (2.16) (d, *J* = 11.6 Hz, 1H, H-6), 2.36 (2.29) (d, *J* = 13.0 Hz, 1H, H_e_-4), 2.75–2.90 (m, 3H, H-9 and H-17), 6.87 (6.82) (s, 1H, H-14), 6.99 (6.95) (d, *J* = 8.0 Hz, 1H, H-12), 7.16 (7.06) (d, *J* = 8.1 Hz, 1H, H-11), 7.46 (7.31) (dd, *J* = 7.8, 4.5 Hz, 1H, H-5′), 7.87 (7.66) (t, *J* = 7.6 Hz, 1H, H-4′), 8.23 (8.38) (d, *J* = 7.6 Hz, 1H, H-3′), 8.54 (8.65) (d, *J* = 4.5 Hz, 1H, H-6′), 8.58 (8.42) (s, 1H, N=CH), 9.16 (9.58) (s, 1H, CONH). ^13^C-NMR (CDCl_3_): 16.3 (15.8), 18.6 (18.4), 21.3 (21.2), 23.9 (23.8) (C-18 and C-19), 25.2 (25.1), 29.7 (29.8), 33.4 (33.2), 37.0 (36.9), 37.1 (37.2), 37.8 (37.7), 45.4 (45.2), 47.3 (47.1), 120.2 (119.8) (C-3′), 123.9 (123.8) (C-12), 124.1 (124.0) (C-11), 124.6 (124.5) (C-5′), 126.8 (126.7) (C-14), 134.6 (134.5) (C-8), 137.5 (137.3) (C-4′), 145.8 (145.7) (C-13), 146.2 (146.4) (C=N), 146.6 (146.5) (C-7), 149.8 (149.5) (C-6′), 153.7 (153.8) (C-2′), 175.7 (176.0) (C=O). MS (ESI) *m*/*z* 404 ([M+H]^+^). Anal. Calcd for C_26_H_33_N_3_O: C, 77.38; H, 8.24, N, 10.41; found C, 77.48; H, 8.11; N, 10.32. 

### 3.4. Antibacterial Studies

The antibacterial activities of the newly synthesized compounds **4a**–**q** were evaluated against four test bacteria: *Bacillus subtilis* CGMCC1.3343, *Escherichia coli* CGMCC1.3373, *Pseudomonas fluorescens* CGMCC1.1802 and *Staphylococcus aureus* CGMCC1.2465. The four bacteria were obtained from China General Microbiological Culture Collection Center (CGMCC), China. The antibacterial activity was assessed in terms of minimum inhibitory concentrations (MICs) by a modified microdilution method [22]. Compounds were dissolved in DMSO and serial double dilutions of each compound (50 μL) were prepared in 96 well micro-trays. The same amount of test microorganism suspension in Martin’s broth (~10^5^ colony forming unit (CFU)/mL) was added to each well to give final concentrations ranging from 250 to 0.45 μg/mL. Amikacin sulfate was co-assayed as positive control, and DMSO was used as negative control. After incubation at 37 ′C for 24 h, the trays were examined for the growth for the test microorganisms. The MIC was defined as the lowest concentrations of compound at which there was no visual turbidity due to microbial growth. All assays were performed in duplicate.

## 4. Conclusions

In this study, a series of new *N*-acylhydrazone derivatives **4a**–**q** of dehydroabietic acid were synthesized and characterized in approach of new antibacterial agents. All the compounds were evaluated for their *in vitro* antibacterial activities against four bacteria. The MIC results indicated that the activities of the investigated compounds were influenced by the physicochemical properties of the substituent at hydrazone moiety. Compounds with fluoro, chloro and nitro substituents exhibited pronounced antibacterial activities, among which the nitrofuranyl derivative **4p** showed strongest activity against two Gram-positive bacteria. The results highlight these new *N*-acylhydrazone derivatives as potential leads for the further investigation on new antibacterial drug candidates. 
